# A Bifunctional Nuclease Promotes the Infection of Zucchini Yellow Mosaic Virus in Watermelon by Targeting P3

**DOI:** 10.3390/plants13233431

**Published:** 2024-12-06

**Authors:** Baoshan Kang, Lifeng Liu, Liming Liu, Mei Liu, Huijie Wu, Bin Peng, Zhiling Liang, Fengnan Liu, Yaoxing Zang, Qinsheng Gu

**Affiliations:** 1 Henan Key Laboratory of Fruit and Cucurbit Biology, Zhengzhou Fruit Research Institute, Chinese Academy of Agricultural Sciences (CAAS), Zhengzhou 450009, China; kangbaoshan@caas.cn (B.K.); liulifeng@caas.cn (L.L.); liuliming@caas.cn (L.L.); lmvirus@126.com (M.L.); wuhuijie@caas.cn (H.W.); pengbin@caas.cn (B.P.); liangzhiling666@163.com (Z.L.); fn13460566877@163.com (F.L.); z1142032183@126.com (Y.Z.); 2 Zhongyuan Research Center, Chinese Academy of Agricultural Sciences (CAAS), Xinxiang 453500, China

**Keywords:** watermelon, zucchini yellow mosaic virus, P3, bifunctional nuclease, interaction

## Abstract

Potyviral P3 is involved in viral replication, movement, and pathogenicity; however, its biochemical function is unknown. In this study, the P3 of the zucchini yellow mosaic virus (ZYMV) interacted with ClBBD, a protein with high ortholog bifunctional nuclease activity, in watermelon. The binding site was shown via yeast two-hybrid screening and BiFC assay to be located at the N-terminus of P3 rather than P3N-PIPO. ClBBD localized predominantly to the chloroplast and plasma membrane. ZYMV P3 was also present in the nucleus and cytoplasm as aggregates. When co-expressed with P3 in tobacco, ClBBD formed aggregates with P3 in the cytoplasm. The knockdown of *ClBBD* using the VIGS vector pV190 and challenge with ZYMV revealed a positive correlation between viral accumulation and ClBBD expression, indicating that ClBBD reduces the resistance of watermelon to ZYMV. Furtherly, we found that when P3 and ClBBD were transiently co-expressed in tobacco, the level of P3 was significantly higher than that when it was expressed alone or co-expressed with GUS. It inferred that ClBBD may be able to stabilize the expression of P3. Overall, the results suggest that the interaction of P3 with ClBBD promotes virus infection, and ClBBD may be involved in stabilizing the expression level of P3.

## 1. Introduction

Zucchini yellow mosaic virus (ZYMV; genus *Potyvirus*) is a threat to the cucurbits industry worldwide. Infected plants display symptoms including distinctive yellow mosaics on the leaves, stunted growth, and fruit deformation and mottling. It affects both the yield and fruit quality, reducing the yield by up to 95% [1]. ZYMV has a positive-stranded, ~9.6 kb RNA genome encoding a polyprotein precursor that is processed into mature proteins by viral proteases [2,3]. The mature proteins are P1, HC-Pro, P3, 6K1, CI, 6K2, VPg, NIa-Pro, NIb, and CP. An additional polypeptide, P3N-PIPO, is produced by the fusion of P3N with an overlapping open reading frame known as PIPO, which is translated after +2 frameshifting of the P3 cistron [3].

P3 protein may be involved in viral replication, pathogenicity, movement, resistance, and host range [4,5,6,7]. P3 may also function as a virulence determinant [8,9,10,11,12,13]. However, P3 is poorly characterized because of the high sequence variation among *Potyvirus* species, resulting in a lack of easily identifiable motifs [14]. Multiple host proteins participate in the potyvirus infection of plants and interact with viral proteins. For example, soybean mosaic virus (SMV) P3 interacts with eukaryotic elongation factor 1A (eEF1A) and hypersensitive response-like lesion-inducing (GmHRLI) proteins to promote viral pathogenesis [15,16], and turnip mosaic virus (TuMV) P3 interacts with endo-1,3-β-glucanase protein (GmGLU) to promote viral replication and intercellular movement [17]. Other identified interactors of P3 include RuBisCO small subunit RbCS [18] and actin-depolymerizing factor 2 [19], which contribute to symptom development and viral localization and movement. In addition, P3 interacts with the P3N-PIPO and 6K2 viral proteins to recruit other viral replication components for intercellular movement [20], and is an essential component of the viral replication complex [21]. Unlike the interactors mentioned above, Rsv4, which has dsRNase activity, interacts with the P3 of SMV to inhibit SMV replication [22].

Bifunctional nuclease related to basal defense responses (BBDs) is a novel bifunctional nuclease first identified in the wild rice species *Oryza minuta*. This protein has RNase and DNase activities [23], but shares no similarities with nucleases with RNase and/or DNase activities, such as RNS1 (RNases T2 family) and BFN1 (type I nuclease family) in Arabidopsis [24,25]. BBD contains three functional domains—a highly conserved region (HCR) domain and a UV-responsive (UVR) domain for protein–protein or protein–DNA interaction ([26]), and a domain of unknown function 151 (DUF151) with bifunctional nuclease activities [23]. It has an important regulatory function in responding to biotic and abiotic stress. OmBBD and AtBBD1 are involved in the ABA-mediated callose deposition in response to infection by *Botrytis cinereal* [26]. Moreover, AtBBD1 functions as a novel positive regulator of drought responses by upregulating the expression of genes responsive to ABA and drought stress [27]. The bifunctional nuclease (BN2) in tobacco regulates the transcription of the key genes in the RNAi pathway by degrading miRNAs [28].

In this study, we identified a bifunctional nuclease (ClBBD) in watermelon that interacts with ZYMV P3, and determined that P3N, but not P3N-PIPO, is the key binding region. By visualizing the subcellular localization of ClBBD and P3, we found that ClBBD is recruited by P3 to form aggregates. Furtherly, we explored the function of ClBBD during viral infection through repressing its expression.

## 2. Results

### 2.1. ZYMV P3 Protein Interacts with the Host Factor ClBBD

**Figure 1:**
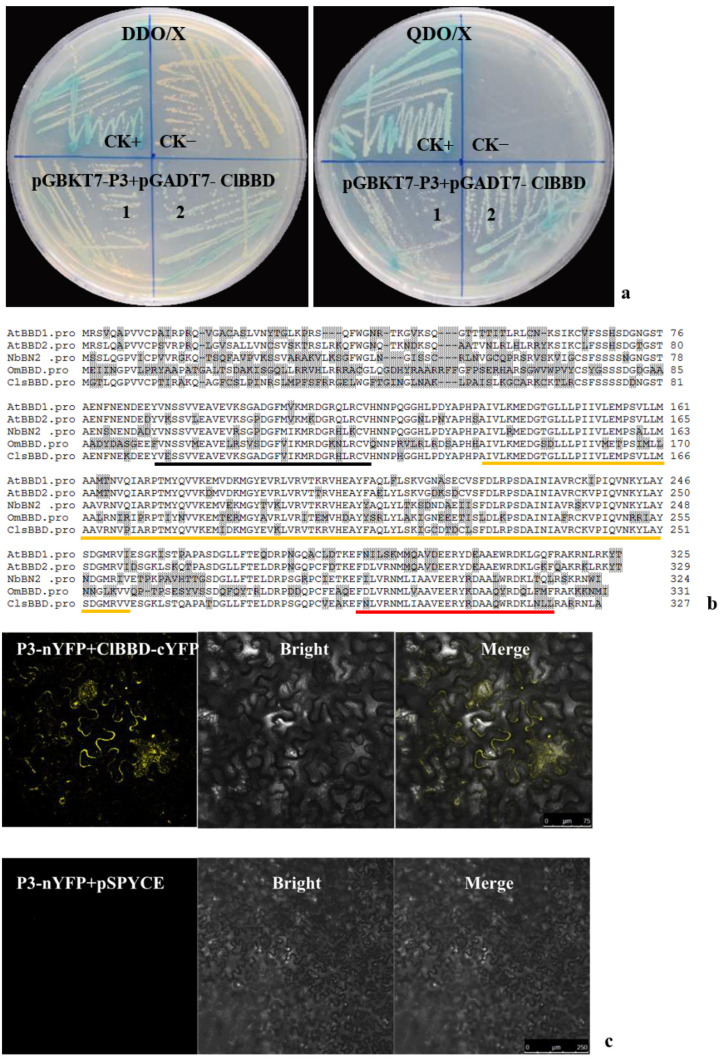

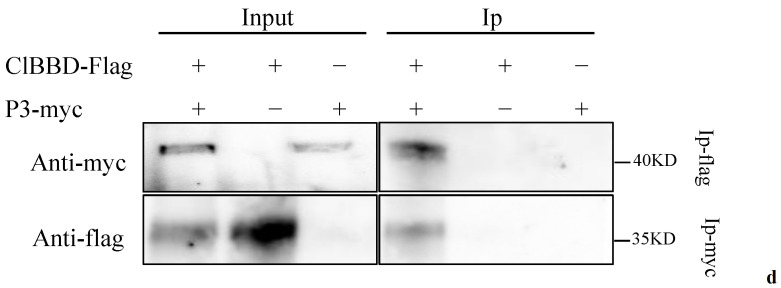
The interaction of ZYMV P3 with ClBBD of watermelon. (**a**) Yeast cells containing pGBKT7-P3 were co-transformed with pGADT7-ClBBD on DDO/X (SD/-Leu/-Trp/X-α-Gal) and QDO/X (SD/-Leu/-Trp/-His/-Ade /x-α-gal) mediums. CK+, positive control for pGADT7-T + pGBKT7-53; CK−, negative control for pGADT7-T + pGBKT7-Lam. (**b**) Alignment of the deduced amino acid sequences of ClBBD with other plant homologs using the Clustal W algorithm. Different amino acids are highlighted in gray. Three domains, a highly conserved region (HCR) domain, an unknown function 151 (DUF151) domain, and a UV-responsive (UVR) domain, are underlined with black, orange, and red colors, respectively. (**c**,**d**) Verification of the interaction between P3 and ClBBD via bimolecular fluorescence complementation assays in *N. benthamiana* and co-immunoprecipitation (Co-IP) assays. YFP fluorescence was detected in the *N. benthamiana* leaves agroinfiltrated with pSPYNE-P3/pSPYCE-ClBBD using pSPYNE-P3/pSPYCE as negative control (**c**). Myc-tagged ZYMV P3 was co-expressed with Flag-tagged ClBBD in *N. benthamiana*. Proteins were immunoprecipitated (Ip) from total extracts using anti-myc or anti-flag antibodies and followed by Western blot using tag-specific antibodies (**d**).

To analyze the functions of P3, interacting host factors were screened via Y2H assay using a watermelon cDNA library and ZYMV P3 as bait. Sequential screenings yielded a single positive clone (Figure 1a). Its nucleotide sequence showed a 99.9% identity with the watermelon gene *Cla97C05G100120*, which was annotated as a bifunctional nuclease. BLAST searches and the alignment of the homologs with *Cla97C05G100120* revealed that their predicted amino acid sequences shared 69.7, 69.1, 59.7, and 69.7% identities, respectively, with AtBBD1 (bifunctional nuclease related to basal defense responses, NP_849890) and AtBBD2 (NP_564093) in *Arabidopsis thaliana*, OmBBD (ABI79452) in *Oryza minuta*, and NbBN2 (MN746382) in *Nicotiana benthamiana* (Figure 1b). *A*. *thaliana* homologs with nuclease activities had no significant conserved residue with BFN (type I nuclease, NP_172585) or RNS1 (S-like RNase1, NP_178399) [24,25]. Also, no homolog of *Cla97C05G100120* was found in the *Citrullus lanatus* genome when the putative amino acid sequence was searched using BLAST. Like other BBDs, the identified protein contained three functional OmBBD or AtBBD domains, an HCR domain, a DUF151 domain, and a UVR domain. Therefore, we designated the protein as ClBBD.

To confirm the interaction between ClBBD and ZYMV P3, bimolecular fluorescence complementation (BiFC) and co-immunoprecipitation (Co-IP) assays were performed. For the BiFC assays, proteins were fused with reciprocal N- or C-terminal halves of yellow fluorescent protein (nYFP and cYFP) and transiently co-expressed in *N*. *benthamiana*. The co-expression of ClBBD with ZYMV P3 resulted in the reconstitution of YFP, indicating a positive interaction (Figure 1c). For Co-IP assays, full-length flag-tagged ClBBD and myc-tagged ZYMV P3 were cloned into the pCAMBIA1300 vector and co-expressed transiently in *N*. *benthamiana* leaves, from which total proteins were extracted at 72 h post-infiltration. The Co-IP of P3-myc with ClBBD-Flag showed a strong signal, whereas the negative control (single expression of ClBBD-Flag or P3-myc) exhibited no signal. A reciprocal experiment supported this result (Figure 1d). Therefore, ZYMV P3 interacts with ClBBD under in vivo conditions.

### 2.2. The Interacting Region of P3 with ClBBD Is at the P3 N-Terminus but Is Not P3N-PIPO

**Figure 2:**
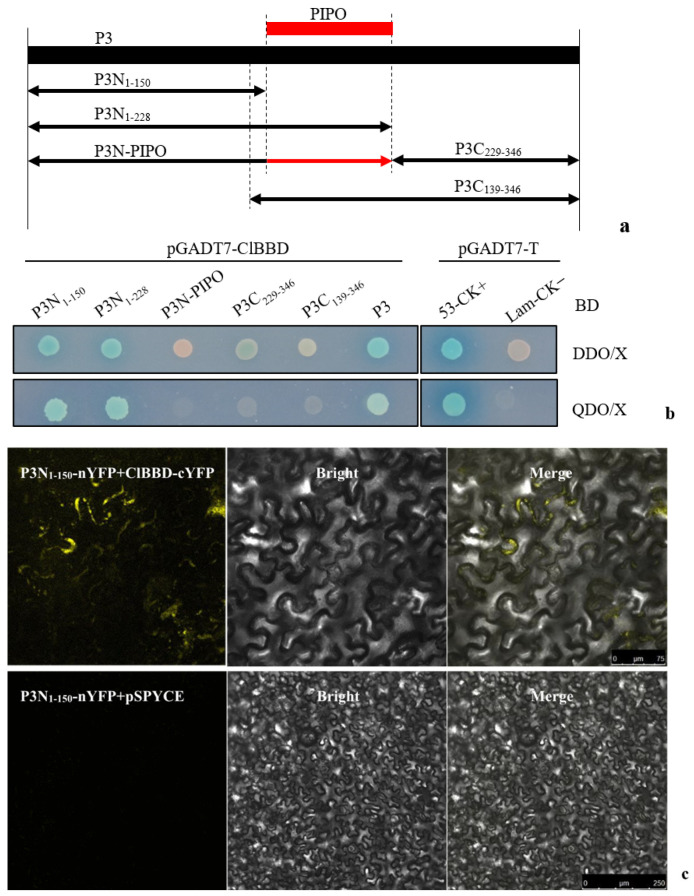
Identification of the key interacting region of P3 with ClBBD via Y2H and BiFC. (**a**) Schematic representations of different parts of P3 and P3N-PIPO. The numbers represent the total number of amino acids contained in the part; (**b**) yeast cells containing different parts of P3 were co-transformed with pGADT7-ClBBD on DDO/X and QDO/X cultures for determining the interacting region with ClBBD; pGADT7-T co-transformed with pGBKT7-53 and pGBKT7-Lam to be as positive control (CK+) and negative control (CK−), respectively; (**c**) confirmation of the interacting region of P3N1–150 via BiFC using pSPYNE-P31–150/pSPYCE as negative control.

To identify the specific region of ZYMV P3 that interacts with ClBBD, we divided the P3 protein into four parts and constructed bait vectors for the N-terminal 1–150 amino acids (aa) (P3N_1–150_) and 1–228 aa (P3N_1–228_), as well as C-terminal 139–346 aa (P3C_139–346_) and 229–346 aa (P3C_229–346_). Additionally, P3N-PIPO was also included (Figure 2a). Y2H assays showed that only yeast cells harboring ClBBD and the P3 N-terminus (P3N_1–150_ and P3N_1–228_) grew on a selective medium (QDO/X). This result suggested that ClBBD protein interacts with the N-terminus (P3N_1–150_ and P3N_1–228_) of P3, but not with its C-terminus (P3C_139–346_ and P3C_229–346_) or P3N-PIPO (Figure 2b). Furthermore, the interacting region of P3N_1–150_ was confirmed via BiFC (Figure 2c). These findings indicate that the N-terminal 1–150 amino acids of P3, specifically, are responsible for its interaction with ClBBD, rather than P3N-PIPO.

### 2.3. P3 Recruits ClBBD in the Cytoplasm

**Figure 3:**
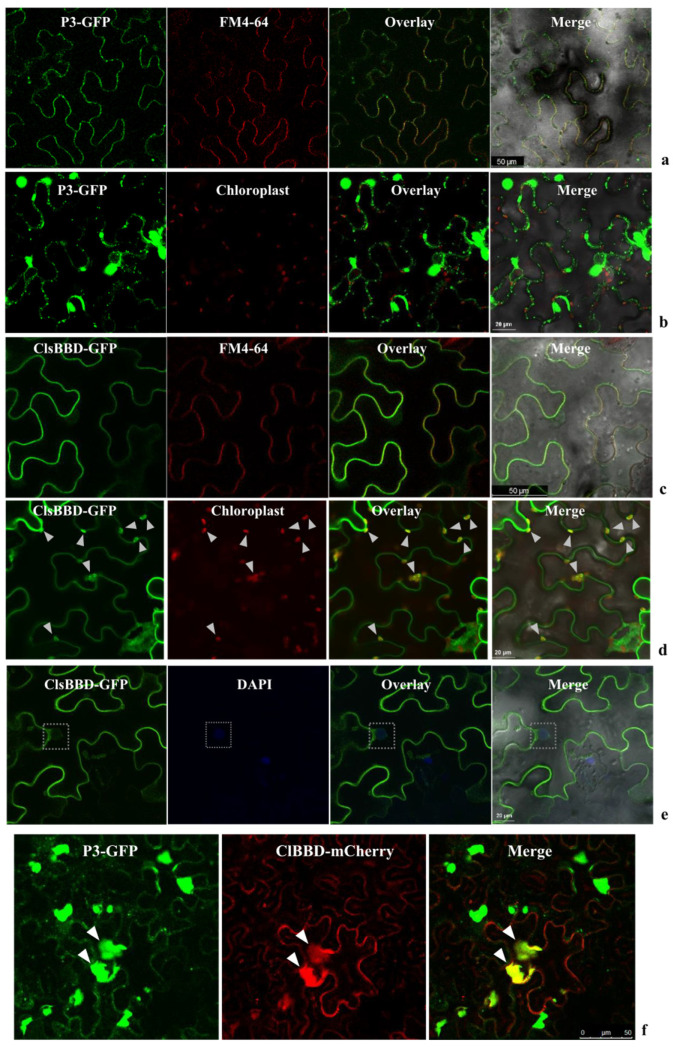
Subcellular localization of ClBBD or ZYMV P3 in *N. benthamiana* leaf epidermal cells. GFP fluorescence is shown as green. The red fluorescence indicates autofluorescence from chloroplasts, mCherry, or FM4-64. The amphiphilic dye FM4-64 and DAPI were used to stain the plasma membrane (PM) and nucleus, respectively. (**a**) P3-GFP showed punctate pattern along the cell wall and overlaid with FM4-64; (**b**) P3-GFP formed irregular aggregates in the cytoplasm and could not overlay with chloroplasts; (**c**,**d**) ClBBD-GFP overlaid with the signal of FM4-64-labeled PM (**c**) and chloroplasts are indicated with white arrows (**d**); (**e**) the weak signal of ClBBD-GFP in nucleus is indicated with white boxes; (**f**) C-terminal GFP/mCherry-fused proteins (P3-GFP, ClBBD-mCherry) were transiently co-expressed in *N. benthamiana* via agroinfiltration. In these cells, ClBBD formed aggregates and was overlaid with P3-GFP (shown by white arrows).

To determine the subcellular localization of ZYMV P3 and ClBBD, the C-termini of both proteins were tagged with GFP or mCherry to be cloned into the pCAMBIA1300 vector, generating constructs for P3-GFP, ClBBD-GFP, and ClBBD-mCherry. These constructs were transiently expressed in *N. benthamiana* leaves. When expressed alone, P3-GFP displayed multiple irregular aggregates in the cytoplasm, with a small portion at the plasma membrane (PM) displaying a punctate pattern along the cell wall (Figure 3a,b). ClBBD-GFP localized predominantly to the PM and chloroplasts, and the nucleus in a small number of cells (Figure 3c–e). Interestingly, when P3-GFP and ClBBD-mCherry were co-expressed, ClBBD-mCherry aggregated with P3-GFP in the cytoplasm (Figure 3f), suggesting the recruitment of ClBBD by P3. These observations provided further evidence for the interaction between P3 and ClBBD.

### 2.4. ClBBD Protein Positively Regulates the Viral Accumulation

**Figure 4:**
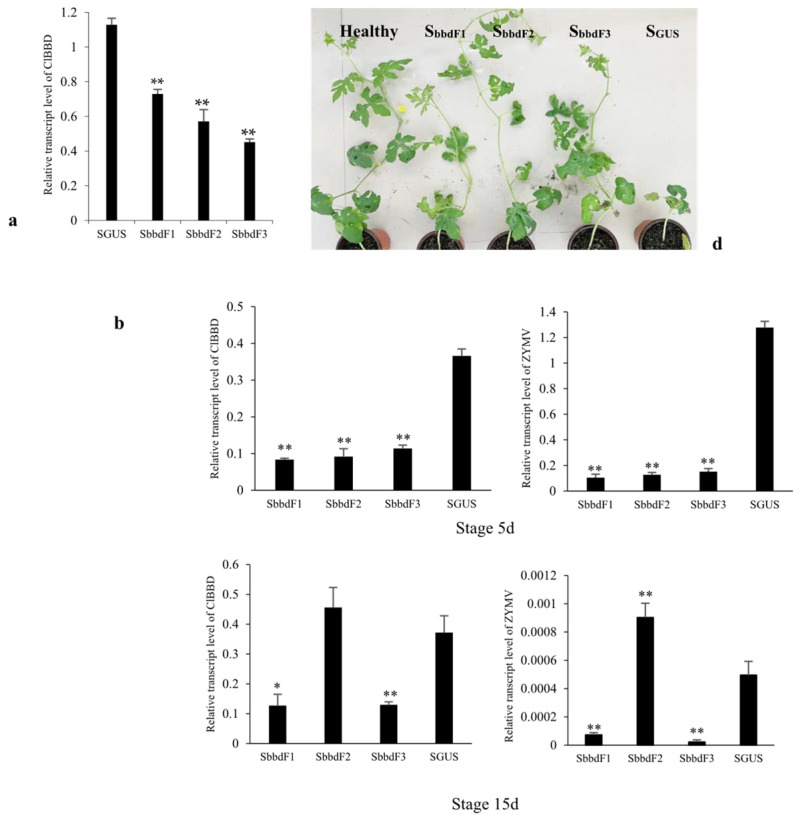

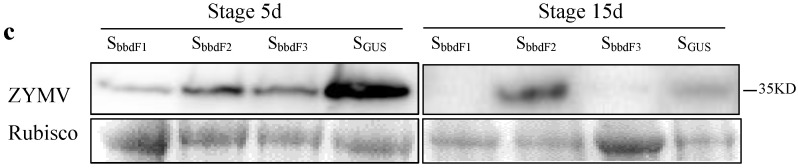
The viral accumulation in watermelon with knockdown of the expression of *ClBBD* via VIGS. (**a**) The mRNA levels of *ClsBBD* in three knockdown lines were significantly reduced at 15 days post-VIGS; (**b**) the mRNA levels of ZYMV and *ClBBD* in three knockdown lines at 10 days post-inoculation with ZYMV at Stages 5d and 15d, respectively; (**c**) detection of the viral accumulation at 10 dpi with ZYMV via Western blot; (**d**) the knockdown plants showed better growth than *S_GUS_* plant at 25 dpi with ZYMV at Stage 5d. Bars represent mean ± SD calculated from three independent biological samples. Asterisks indicate the significant difference to *S_GUS_* control; * and ** represent *p* < 0.05 and 0.01, respectively.

To investigate the contribution of ClBBD to the resistance of watermelon to ZYMV, we used the cucumber green mottle mosaic virus (CGMMV)-based VIGS (virus-induced gene silencing) vector to knock out *ClBBD* expression in watermelon. We generated three constructs (*S_bbdF1_*, *S_bbdF2_*, *S_bbdF3_*) containing different fragments of *Cla97C05G100120* to reduce the transcript level of *ClBBD* in cv. Hongheping. By 15 days post-infiltration, the mRNA level of *ClBBD* was significantly reduced in all the knockdown plants (*S_bbdF1_*, *S_bbdF2_*, *S_bbdF3_*) compared to the control plants infiltrated with the construct containing a 220 bp fragment of *GUS* (*S_GUS_* plant) (Figure 4a). Based on this result, we challenged the inoculation with ZYMV of these knockdown plants at 5 days (Stage 5d) and 15 days (Stage 15d) post-VIGS, respectively, and analyzed the mRNA level of *ClBBD* and viral accumulation at 10 days post-inoculation with ZYMV. When challenged at Stage 5d, the level of *ClBBD* was reduced in all the knockdown plants, and virus accumulation was also markedly lower than in *S_GUS_* plants at 10 dpi. Compared with the control, the knockdown plants had less impact on growth (Figure 4b–d). Similar results were obtained in melon (Supplementary Figure S1). When challenged at Stage 15d, the *ClBBD* expression level was decreased in *S_bbdF1_* and *S_bbdF3_* plants, in which the ZYMV accumulation was significantly lower than in the *S_GUS_* plants. However, in the *S_bbdF2_* plants, the *ClBBD* expression level was not lower than in the *S_GUS_* plants, suggesting the failure of silencing currently, and the silencing duration of *S_bbdF2_* was shorter than for *S_bbdF1_* and *S_bbdF3_*. This may depend on the fragment inserted into the VIGS vector. Correspondingly, the viral accumulation of the *S_bbdF2_* plants was at a high level (Figure 4b,c). It suggested that ClBBD positively regulates the viral accumulation.

### 2.5. ClBBD Is Involved in Regulating the Expression of P3

**Figure 5:**
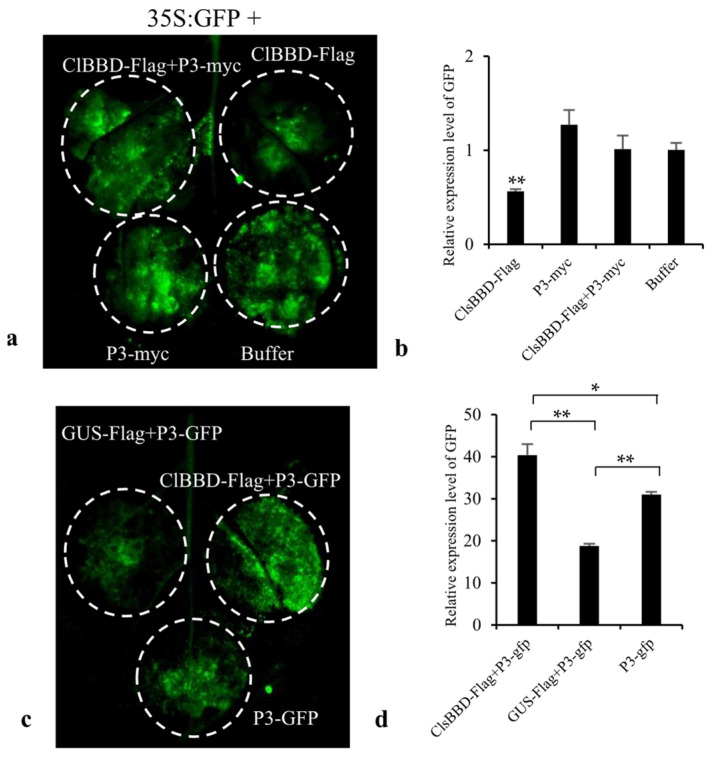

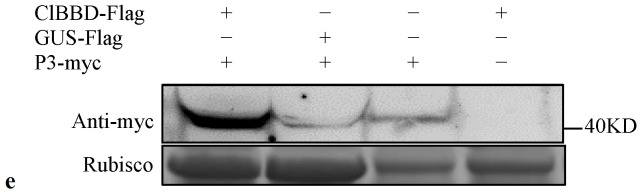
The test of ClBBD regulating the expression of P3. (**a**) Transient co-expression of ClBBD-Flag, ClBBD-Flag plus P3-myc, P3-myc, or Buffer with 35S:GFP in *N. benthamiana*. (**b**) RT-qPCR analysis of relative *GFP* mRNA levels. (**c**) Transient co-expression of ClBBD-Flag or GUS-Flag with P3-GFP, and only P3-GFP in *N. benthamiana*. (**d**) qRT-PCR analysis of relative *P3-GFP* mRNA levels. (**e**) Western blot assay of the P3 protein level through expressing transiently of P3-myc alone, or co-expression of P3-myc with ClBBD-Flag, or co-expression of P3-myc with GUS-Flag in *N. benthamiana*. The constructs GUS-Flag, ClBBD-Flag, P3-GFP and P3-myc were generated using the pCAMBIA1300 vector, and 35S:GFP was constructed using PCB301 vector. Bars represent mean ± SD calculated from three independent biological samples. Asterisks indicate the significant difference; * and ** represent *p* < 0.05 and 0.01, respectively.

We speculated that ClBBD is involved in host RNAi based on the function of the bifunctional nuclease-2 (BN2) in *N. benthamiana*, which is a BBD homolog, degrading microRNAs post-transcriptionally and indirectly regulating the level of mRNA encoding the key components of RNAi in plants [28]. To explore the function of ClBBD, we transiently co-expressed 35S:GFP with ClBBD-Flag, ClBBD-Flag plus P3-myc, and P3-myc, respectively, in *N. benthamiana*. At four days post-agroinoculation, leaves overexpressing ClBBD-Flag exhibited weaker GFP fluorescence and lower *GFP* mRNA levels compared to those expressing 35S:GFP alone. However, when 35S:GFP was co-expressed with ClBBD-Flag plus P3-myc, the GFP fluorescence intensities and mRNA levels were similar to those in the 35S:GFP control (Figure 5a,b). These findings inferred that ClBBD enhances the efficiency of RNAi, an effect that is partially attenuated by its interaction with P3.

To investigate this effect in relation to the interaction with P3, we transiently co-expressed P3-GFP with ClBBD-Flag or a control gene (GUS-Flag) in *N. benthamiana*. Our results indicated that the co-expression of ClBBD-Flag with P3-GFP resulted in a significantly higher fluorescence intensity of P3-GFP compared to other cases, including the co-expression of GUS-Flag with P3-GFP or P3-GFP alone expression (Figure 5c). This observation is supported by the mRNA levels of *GFP*, which align with the fluorescence intensity (Figure 5d). To validate our observation at the protein level, we co-expressed the myc-tagged P3 with ClBBD-Flag or GUS-Flag. The protein level of P3 was found to be higher when co-expressed with ClBBD-Flag compared to its co-expression with GUS-Flag or expression alone (Figure 5e). This result was also in accordance with the result of VIGS. This suggested that ClBBD plays a role in maintaining the stability or expression level of P3, possibly by mitigating the host antiviral RNAi defense.

## 3. Discussion

Few studies have focused on BBDs; consequently, their function(s) are unclear. ClBBD contains a DUF151 domain, also known as a bifunctional nuclease (BFN) domain or DNase-RNase domain [26]. Several studies reported that BBDs have non-substrate-specific DNase and RNase activities in vitro [23,26], and dsRNA is the preferred substrate for DNA/RNA non-specific nucleases [29]. However, our experiment showed that ClBBD did not exhibit activity toward total RNA or dsRNA in vitro. This may imply that the nuclease substrate of ClBBD is not non-specific. BN2, a homolog of BBD in *N. benthamiana*, had no effect on ssDNA, pre-miR168, or long RNAs, and degraded mature miRNAs in a non-specific manner in vitro [28]. Although AtBBD1, AtBBD2, OmBBD, and BN2 contain a conserved bifunctional nuclease domain, they showed different substrate specificities in vitro. The discrepancy may be caused by differences in amino-acid sequences, reaction conditions, or activities in vitro after purification as a result of their high insolubility [30]. Although their substrates vary, the biological functions of nucleases are directly or indirectly related to the RNAi pathway. BN2 participates in the RNAi pathway by indirectly regulating the level of DCL1 or AGO1/2 mRNA via the degradation of their miRNAs. In some insects, nucleases inhibit RNAi by cleaving dsRNA [30,31,32]. Interestingly, ribonucleases (RNS1 and RNS3) with RNase 1 activities regulate RNAi by cleaving tRNAs to generate tRNA-derived fragments (tRFs), some of which are associated with ARGONAUTE (AGO) proteins [33,34,35]. Our results showed that ClBBD overexpression promotes RNAi in *N. benthamiana*, indicating that ClBBD may be related to the pathway of RNAi. However, when co-expressed with P3, ClBBD can stabilize the expression of P3. There are two possible reasons; on the one hand, the binding of P3 and ClBBD may decrease the free ClBBD level, thereby decreasing RNAi efficiency. On the other hand, the conformation of ClBBD may be altered by its interaction with P3, leading to changes in the nuclease substrate, which may affect the host RNAi. If the decrease in RNAi efficiency is caused solely by a reduction in free ClBBD, the protein level of P3-GFP co-expressed with ClBBD-Flag in leaves should be similar or slightly lower than P3-GFP expression alone. However, several replicate experiments yielded the same result—the level of P3-GFP when co-expressed with ClBBD-Flag in leaves was significantly higher than that in leaves in which it was expressed alone or with GUS-Flag (Figure 5). Therefore, we speculated that ClBBD promotes viral infection by inhibiting P3 silencing via its interaction with P3. This was supported by the results of the VIGS knockdown of ClBBD. In addition, AtBBD1 has been reported to localize in nucleus and regulate the expression of key regulatory genes in the ABA-signaling [26,27]. Our results also showed that ClBBD-GFP fusion proteins were localized in the nuclei of tobacco. Does this indicate that ClBBD can also bind DNA to function as a transcription factor? If so, ClBBD may also exert its effects by regulating the expression of other genes.

P3 is one of two membrane proteins encoded by potyviruses, and during infection is involved in viral replication, movement, and symptom expression [4,13,21]. These processes involve multiple organelles, such as the endoplasmic reticulum (ER), PM, and nucleus, which are linked to viral intercellular movement and the host defense response [16,17]. In this study, P3 formed aggregates mainly in the cytoplasm, where P3 forms complexes with other viral proteins and is responsible for the intercellular transport of the viral genome [20]. ClBBD is mainly localized in chloroplasts, the sites of replication for several plant viruses [4,36]. P3 is an essential component of the virus replication complex (VRC) [20,21], and targeting ClBBD may be an important step in viral replication. The interaction between ClBBD and P3 may reduce the host RNAi-mediated degradation of P3, enabling it to perform its functions in replication and mobility. In soybean, the host factor Rsv4 enters the viral replication compartment by binding with SMV P3. Interestingly, Rsv4 is a dsRNase that degrades viral dsRNA to inhibit viral replication [22]. Our results indicate that P3 has multiple functions in virus infection, and the functions of nucleases depend on their substrates. Although doing so is challenging, the substrates of ClBBD need to be identified to provide insight into its function.

## 4. Conclusions

ClBBD interacts with ZYMV P3 in *C. lanatus* via the P3 N-terminus rather than P3N-PIPO. Transient expression showed that ClBBD localized mainly in chloroplasts and the PM, but in the presence of P3, it could be recruited in the cytoplasm. The knockdown of its expression revealed that ClBBD positively regulates the viral accumulation in watermelon. The role of ClBBD may be associated with regulating the expression level of P3. These findings suggest that the interaction of P3N with ClBBD promotes viral infection.

## 5. Experimental Procedures

### 5.1. Plant Material and Virus

All the plants used in this study were grown in 10 cm pots with a mixture of peat and vermiculite (3:1). *Nicotiana benthamiana* plants were grown and kept in growth chambers at 23 °C/22 °C under a 16 h/8 h light/dark cycle for transient expression. Watermelon (*Citrullus lanatus*) cv. “Hongheping” plants were grown and maintained in growth chambers at 27 °C/23 °C under a 16 h/8 h light/dark cycle for viral inoculation. The genomic clones of ZYMV-CH87 isolate which originated from a naturally infected melon have been described previously (Liu et al. 2021, [37]).

### 5.2. Constructs

For the yeast two-hybrid constructs, the coding sequences of P3, P3N_1–150_, P3N_1–228_, P3C_229–346_, and P3C_139–346_ were amplified from ZYMV-CH87 using specific primers. Each coding sequence was introduced with an artificial ATG start codon and TAG stop codon at the 5′ and 3′ ends, respectively. P3N-PIPO was produced by one nucleotide insertion in the putative frameshift site (AGAAAAAA) to allow the expression of P3N-PIPO without frameshifting. The bait constructs were generated by transferring the corresponding coding sequences into the NcoI-BamHI sites of pGBKT7. To construct the prey construct, the ClBBD coding sequence was amplified using the cDNA of watermelon as a template and cloned into the EcoRI-BamHI sites of pGADT7 (Clontech).

For the BiFC constructs expressing P3-nYFP, P3N_1–150_-nYFP, and ClBBD-cYFP, each corresponding sequence was amplified and cloned into the BamHI-XhoI sites of the pSPYNE or pSPYCE vector, as described [38].

For the subcellular localization and transient expression studies, we used the pCAMBIA1300 vector expressing ClBBD-GFP, P3-GFP, ClBBD-mCherry, ClBBD-Flag, P3-myc, and GUS-Flag.

All the constructs were verified via DNA sequencing.

### 5.3. Yeast Two-Hybrid Assay

A watermelon leaf-specific cDNA library from ZYMV-infected watermelon (cv. H1) was cloned into the vector pGADT7 following the procedures in the Make Your Own “Make & Plate” Library System User Manual (Clontech). Library screening was performed according to the Matchmaker Gold Yeast Two-Hybrid System User Manual (Clontech). From the yeast cells grown on a high-stringency quadruple dropout medium (SD/-Leu/-Trp/-Ade/-His, QDO), prey plasmids were rescued via *E. coli* and sequenced. The sequences of DNA and protein were analyzed using the BLAST algorithms on the NCBI and CuGenDBv2 websites.

To confirm the interaction in yeast, the bait and pray plasmids containing full coding sequences were co-transformed into the cell of Y2HGold using the yeast maker, Yeast Transformation System 2 (Clontech). Co-transformants were initially plated on a double dropout medium containing X-α-Gal (SD/-Leu/-Trp/+X-a-Gal, DDO/X), and positive yeast colonies were subsequently transferred onto QDO medium plus X-α-Gal (QDO/X). Plasmid pGADT7-T was co-transformed with pGBKT7-53 as a positive control, and with pGBKT7-Lam as a negative control.

### 5.4. Agroinfiltration in N. benthamiana and Watermelon

The experiments of BiFC, subcellular localization, and overexpression were performed using 3–4-week-old *N. benthamiana* plants. The VIGS and virus infection were carried out using watermelon plants. Briefly, independent cultures of *A. tumefaciens* GV3101 carrying recombinant plasmids were cultured for 12–24 h in LB liquid medium plus the appropriate antibiotics. The cultures were centrifugated and then resuspended in an infiltration medium containing 10 mM MgCl_2_, 10 mM MES pH 5.6, and 150 mM acetosyringone, and incubated for 2 h at room temperature for infiltration. The density of the agrobacterium was adjusted to OD600 ≈ 0. 5 for the experiments in *N. benthamiana*, and OD600 ≈ 1.0 for the experiments in watermelon.

### 5.5. Co-Immunoprecipitation Assays

The proteins of P3-myc and ClBBD-flag were expressed transiently in the youngest fully-expanded leaves of 3-week-old *N. benthamiana* plants via agroinfiltration. The total proteins were extracted from the infiltrated leaves at 72 h post-infiltration in buffer containing 50 mM Tris acetate pH 7.5, 150 mM NaCl, 10 mM NaF, 0.5 mM DTT, 1 mM PMSF, a complete protease inhibitor cocktail, and 0.1% Triton X-100. The protein extract after centrifugation was prepared for immunoprecipitation using Anti-Myc tag antibody (Agarose, Abcam, Cambridge, UK) or DYKDDDDK-tagged Rabbit monoclonal antibody (mAb) (Sepharose Bead Conjugate, CST, Danvers, MA, USA). Twenty microliters of anti-tag microbeads and 400 μL of protein extracts were mixed and incubated overnight at 4 °C with gentle shaking. The microbeads were washed five times with immunoprecipitation lysis/wash buffer and eluted with elution buffer (Thermo Scientific Pierce, Waltham, MA, USA). The eluted samples were recovered in nonreducing sample buffer (Thermo Scientific Pierce) via brief boiling and analyzed via Western blotting using anti-Myc (CST) and anti-FLAG antibodies (Sigma-Aldrich, St. Louis, MO, USA). The Western blotting was performed as described by [39].

### 5.6. Confocal Microscopy

Confocal laser scanning microscopy was performed using a Leica TCS SP5 microscope (Leica, Wetzlar, Germany) and Olympus FV3000 microscope (Olympus, Hamburg, Germany). A patch of the leaf was cut from the infiltrated area and the corresponding fluorescence was visualized using the confocal laser scanning microscope (Leica) at 36–72 h post-agroinfiltration. The plasma membrane (PM) was visualized via FM4-64 staining [40]. Before the confocal microscopy, the leaves were immersed in 5 µM FM4-64 solution and kept on ice for 5 min. The nucleus was visualized via DAPI staining. The excitation laser wavelengths for DAPI, GFP, YFP, and mCherry/FM4-64 were 358, 488, 510, and 568 nm, respectively. The emission bandwidths of 450 to 488 nm (for DAPI), 500 to 550 nm (for GFP), 520 to 550 nm (for YFP), and 570 to 620 nm (for mCherry or FM4-64) were collected. To avoid signal cross-contamination, each fluorescence signal was collected separately when multiple fluorescence signals needed to be recorded.

### 5.7. Virus-Induced Gene Silencing (VIGS) Assay and Viral Infections

VIGS assays were performed using the cucumber green mottle mosaic virus (CGMMV)-based VIGS vector as described by Liu et al. [41]. Three different fragments of *ClBBD*, 213 bp, 319 bp, and 228 bp in size were amplified using special primers and cloned into the BamHI site of the VIGS vector pV190, respectively, generating three pV190 constructs (S_bbdF1_, S_bbdF2_, and S_bbdF3_). The two cotyledons of watermelon cv. Hongheping plants were infiltrated with *A. tumefaciens* GV3101 carrying pV190-based constructs at the one true-leaf stage. Systemic leaves were collected to analyze the expressional level of the target genes. Each vector of VIGS infiltrated in at least 6 plants. The pV190 vector containing a ~250 bp fragment of *GUS* gene was used as a control.

Viral infections were performed using a ZYMV infectious clone via Agrobacterium. Watermelon cv. Hongheping plants were used to inoculate ZYMV through infiltration when the *ClBBD* gene was suppressed for 5 days (Stage 5d) and 15 days (Stage 15d), respectively. Systemic leaves were collected to analyze the viral accumulation at 10 days post-inoculation with ZYMV.

### 5.8. RNA Extraction and Quantitative Real-Time PCR Analysis

The total RNA was extracted from the new leaves of watermelon plants or inoculated leaves of tobacco using the RNA extract kit (Tiangen, Beijing, China). First-strand cDNA synthesis was carried out using a FastKing RT Kit (Tiangen). Quantitative real-time PCR was performed in a LightCycler 480 instrument (Roche, Munich, Germany) using FS Universal SYBR Green Master (Roche), with cycling conditions according to the manufacturer’s instructions. Briefly, qRT-PCR was conducted in a 20 μL reaction volume consisting of 1 μL of cDNA template, 1 μL each of forward and reverse primers, 10 μL of 2× Master Mix, and 8 μL of ddH_2_O. The reaction was conducted using a program consisting of 95 °C for 5 min, 40 cycles of 15 s at 95 °C, 20 s at 60 °C, and 20 s at 72 °C, and fluorescence acquisition at 72 °C. The primers were designed according to the database of watermelon (97103) v2 to amplify the gene-specific PCR products of 150–300 bp in length. To normalize the cDNA, the *tubulin* gene (*Cla97C02G050600*) and the *actin* gene (XM_016619439) were used as an internal control for the watermelon and tobacco samples, respectively. The gene expression was quantified using the relative quantification method in at least three independent biological replicates for each sample, and the standard deviation (SD) values are shown.

### 5.9. Statistical Analyses

Data are shown as the means ± SD. The statistical significance was determined via the two-tailed Student’s *t*-test using SPSS 16.0 software. Variations were considered statistically significant if the *p* value < 0.05 (* *p* < 0.05; ** *p* < 0.01).

## Data Availability

The data that support the findings of this study are available from the corresponding author upon reasonable request.

## Supplementary Materials

The following supporting information can be downloaded at https://www.mdpi.com/article/10.3390/plants13233431/s1, Supplementary Figure S1. The viral accumulation in melon with knockdown of the expression of *CmBBD* via VIGS. (a) The mRNA level of *CmBBD* was repressed in melon at 13 days post-infiltration with S_bbdF2_ VIGS constructs; (b) when challenging inoculation with ZYMV-eGFP at Stage 5d, the eGFP fluorescence in S_bbdF2_ line was weaker than that in *S_GUS_* or *S_PDS_* control at 8dpi. Asterisks indicate the significant difference to *S_GUS_* control; ** represent *p* < 0.01. Supplementary Figure S2. Ribonuclease activity assays on dsRNA and total RNA in vitro. RNA were incubated in buffer 1 or buffer 2 alone (−), or with about 2.1 μg purified ClBBD-His (+), at 25 °C for 2 h. DsGFP-1 was synthesized in vitro using MEGAscript RNAi Kit and dsGFP-2 was expressed in *E. coli*-HT115 and then was extracted using Total RNA Extraction Kit. Total RNA was extracted from the leaves of watermelon. Reaction Buffer 1 was referred to Wang et al. [28], and reaction buffer 2 was referred to Huque et al. [27]. The red arrows pointed to the dsGFP-2.

## References

[1] Desbiez C., Lecoq H. (1997). Zucchini yellow mosaic virus. Plant Pathol..

[2] Urcuqui-Inchima S., Haenni A.L., Bernardi F. (2001). Potyvirus proteins: A wealth of functions. Virus Res..

[3] Chung B.Y., Miller W.A., Atkins J.F., Firth A.E. (2008). An overlapping essential gene in the Potyviridae. Proc. Natl. Acad. Sci. USA.

[4] Cui X., Wei T., Chowda-Reddy R.V., Sun G., Wang A. (2010). The Tobacco etch virus P3 protein forms mobile inclusions via the early secretory pathway and traffics along actin microfilaments. Virology.

[5] Johansen I.E., Lund O.S., Hjulsager C.K., Laursen J. (2001). Recessive resistance in Pisum sativum and potyvirus pathotype resolved in a gene-for-cistron correspondence between host and virus. J. Virol..

[6] Merits A., Guo D., Jarvekulg L., Saarma M. (1999). Biochemical and genetic evidence for interactions between potato A potyvirus-encoded proteins P1 and P3 and proteins of the putative replication complex. Virology.

[7] Jenner C.E., Wang X., Tomimura K., Ohshima K., Ponz F., Walsh J.A. (2003). The dual role of the potyvirus P3 protein of Turnip mosaic virus as a symptom and avirulence determinant in brassicas. Mol. Plant-Microbe Interact. MPMI.

[8] Choi S.H., Hagiwara-Komoda Y., Nakahara K.S., Atsumi G., Shimada R., Hisa Y., Naito S., Uyeda I. (2013). Quantitative and qualitative involvement of P3N-PIPO in overcoming recessive resistance against Clover yellow vein virus in pea carrying the cyv1 gene. J. Virol..

[9] Hajimorad M.R., Eggenberger A.L., Hill J.H. (2008). Adaptation of Soybean mosaic virus avirulent chimeras containing P3 sequences from virulent strains to Rsv1-genotype soybeans is mediated by mutations in HC-Pro. Mol. Plant-Microbe Interact. MPMI.

[10] Chowda-Reddy R.V., Sun H., Chen H., Poysa V., Ling H., Gijzen M., Wang A. (2011). Mutations in the P3 protein of Soybean mosaic virus G2 isolates determine virulence on Rsv4-genotype soybean. Mol. Plant-Microbe Interact. MPMI.

[11] Wang Y., Khatabi B., Hajimorad M.R. (2015). Amino acid substitution in P3 of Soybean mosaic virus to convert avirulence to virulence on Rsv4-genotype soybean is influenced by the genetic composition of P3. Mol. Plant Pathol..

[12] Wen R.H., Maroof M.A., Hajimorad M.R. (2011). Amino acid changes in P3, and not the overlapping pipo-encoded protein, determine virulence of soybean mosaic virus on functionally immune Rsv1-genotype soybean. Mol. Plant Pathol..

[13] Desbiez C., Gal-On A., Girard M., Wipf-Scheibel C., Lecoq H. (2003). Increase in Zucchini yellow mosaic virus Symptom Severity in Tolerant Zucchini Cultivars Is Related to a Point Mutation in P3 Protein and Is Associated with a Loss of Relative Fitness on Susceptible Plants. Phytopathology.

[14] Eiamtanasate S., Juricek M., Yap Y.K. (2007). C-terminal hydrophobic region leads PRSV P3 protein to endoplasmic reticulum. Virus Genes..

[15] Luan H., Shine M.B., Cui X., Chen X., Ma N., Kachroo P., Zhi H., Kachroo A. (2016). The Potyviral P3 Protein Targets Eukaryotic Elongation Factor 1A to Promote the Unfolded Protein Response and Viral Pathogenesis. Plant Physiol..

[16] Luan H., Liao W., Niu H., Cui X., Chen X., Zhi H. (2019). Comprehensive Analysis of Soybean Mosaic Virus P3 Protein Interactors and Hypersensitive Response-Like Lesion-Inducing Protein Function. Int. J. Mol. Sci..

[17] Shi F., Wang Y., Zhang F., Yuan X., Chen H., Chen X., Chen X., Cui X. (2020). Soybean Endo-1,3-Beta-Glucanase (GmGLU) Interaction with Soybean mosaic virus-Encoded P3 Protein May Contribute to the Intercelluar Movement. Front. Genet..

[18] Lin L., Luo Z., Yan F., Lu Y., Zheng H., Chen J. (2011). Interaction between potyvirus P3 and ribulose-1,5-bisphosphate carboxylase/oxygenase (RubisCO) of host plants. Virus Genes..

[19] Lu L., Wu G., Xu X., Luan H., Zhi H., Cui J., Cui X., Chen X. (2015). Soybean actin-depolymerizing factor 2 interacts with Soybean mosaic virus-encoded P3 protein. Virus Genes..

[20] Chai M., Wu X., Liu J., Fang Y., Luan Y., Cui X., Zhou X., Wang A., Cheng X. (2020). P3N-PIPO Interacts with P3 via the Shared N-Terminal Domain To Recruit Viral Replication Vesicles for Cell-to-Cell Movement. J. Virol..

[21] Cui X., Yaghmaiean H., Wu G., Wu X., Chen X., Thorn G., Wang A. (2017). The C-terminal region of the Turnip mosaic virus P3 protein is essential for viral infection via targeting P3 to the viral replication complex. Virology.

[22] Ishibashi K., Saruta M., Shimizu T., Shu M., Anai T., Komatsu K., Yamada N., Katayose Y., Ishikawa M., Ishimoto M. (2019). Soybean antiviral immunity conferred by dsRNase targets the viral replication complex. Nat. Commun..

[23] Huque A., So W.M., You M.K., Shin J.S. (2020). Phylogenetic Analysis and In Vitro Bifunctional Nuclease Assay of Arabidopsis BBD1 and BBD2. Molecules.

[24] LeBrasseur N.D., MacIntosh G.C., Perez-Amador M.A., Saitoh M., Green P.J. (2002). Local and systemic wound-induction of RNase and nuclease activities in Arabidopsis: RNS1 as a marker for a JA-independent systemic signaling pathway. Plant J..

[25] Perez-Amador M.A., Abler M.L., De Rocher E.J., Thompson D.M., van Hoof A., LeBrasseur N.D., Lers A., Green P.J. (2000). Identification of BFN1, a bifunctional nuclease induced during leaf and stem senescence in Arabidopsis. Plant Physiol..

[26] You M.K., Shin H.Y., Kim Y.J., Ok S.H., Cho S.K., Jeung J.U., Yoo S.D., Kim J.K., Shin J.S. (2010). Novel bifunctional nucleases, OmBBD and AtBBD1, are involved in abscisic acid-mediated callose deposition in Arabidopsis. Plant Physiol..

[27] Huque A., So W., Noh M., You M.K., Shin J.S. (2021). Overexpression of AtBBD1, Arabidopsis Bifunctional Nuclease, Confers Drought Tolerance by Enhancing the Expression of Regulatory Genes in ABA-Mediated Drought Stress Signaling. Int. J. Mol. Sci..

[28] Wang Y., Gong Q., Wu Y., Huang F., Ismayil A., Zhang D., Li H., Gu H., Ludman M., Fatyol K. (2021). A calmodulin-binding transcription factor links calcium signaling to antiviral RNAi defense in plants. Cell Host Microbe.

[29] Meiss G., Gast F.U., Pingoud A.M. (1999). The DNA/RNA non-specific Serratia nuclease prefers double-stranded A-form nucleic acids as substrates. J. Mol. Biol..

[30] Guan R.B., Li H.C., Fan Y.J., Hu S.R., Christiaens O., Smagghe G., Miao X.X. (2018). A nuclease specific to lepidopteran insects suppresses RNAi. J. Biol. Chem..

[31] Prentice K., Smagghe G., Gheysen G., Christiaens O. (2019). Nuclease activity decreases the RNAi response in the sweetpotato weevil Cylas puncticollis. Insect Biochem. Mol. Biol..

[32] Li J., Du J., Li S., Wang X. (2022). Identification and Characterization of a Double-Stranded RNA Degrading Nuclease Influencing RNAi Efficiency in the Rice Leaf Folder Cnaphalocrocis medinalis. Int. J. Mol. Sci..

[33] Megel C., Hummel G., Lalande S., Ubrig E., Cognat V., Morelle G., Salinas-Giege T., Duchene A.M., Marechal-Drouard L. (2019). Plant RNases T2, but not Dicer-like proteins, are major players of tRNA-derived fragments biogenesis. Nucleic Acids Res..

[34] Gu H., Lian B., Yuan Y., Kong C., Li Y., Liu C., Qi Y. (2022). A 5’ tRNA-Ala-derived small RNA regulates anti-fungal defense in plants. Sci. China Life Sci..

[35] Cognat V., Morelle G., Megel C., Lalande S., Molinier J., Vincent T., Small I., Duchene A.M., Marechal-Drouard L. (2017). The nuclear and organellar tRNA-derived RNA fragment population in Arabidopsis thaliana is highly dynamic. Nucleic Acids Res..

[36] Wei T., Zhang C., Hou X., Sanfacon H., Wang A. (2013). The SNARE protein Syp71 is essential for turnip mosaic virus infection by mediating fusion of virus-induced vesicles with chloroplasts. PLoS Pathog..

[37] Liu L.M., Kang B.S., Peng B., Wu H.J., Su Y.D., Gu Q.S. (2021). Construction of ZYMV infectious clone carrying eGFP and its infectivity. Acta Phytopathol. Sin..

[38] Walter M., Chaban C., Schutze K., Batistic O., Weckermann K., Nake C., Blazevic D., Grefen C., Schumacher K., Oecking C. (2004). Visualization of protein interactions in living plant cells using bimolecular fluorescence complementation. Plant J..

[39] Lough T.J., Balmori E., Beck D.L., Forster R.L. (1998). Western analysis of transgenic plants. Methods Mol. Biol..

[40] Fischer-Parton S., Parton R.M., Hickey P.C., Dijksterhuis J., Atkinson H.A., Read N.D. (2000). Confocal microscopy of FM4-64 as a tool for analysing endocytosis and vesicle trafficking in living fungal hyphae. J. Microsc..

[41] Liu M., Liang Z.L., Aranda M.A., Hong N., Liu L.M., Kang B.S., Gu Q.S. (2020). A cucumber green mottle mosaic virus vector for virus-induced gene silencing in cucurbit plants. Plant Methods.

