# Supplementary material for: A Bifunctional Nuclease Promotes the Infection of Zucchini Yellow Mosaic Virus in Watermelon by Targeting P3
Source: Plants (Basel). 2024 Dec 6;13(23):3431. doi: 10.3390/plants13233431 (PMC11644367; doi:10.3390/plants13233431)
Supplement: Supplementary file 1 [file plants-13-03431-s001.zip › Figure S1.pdf]

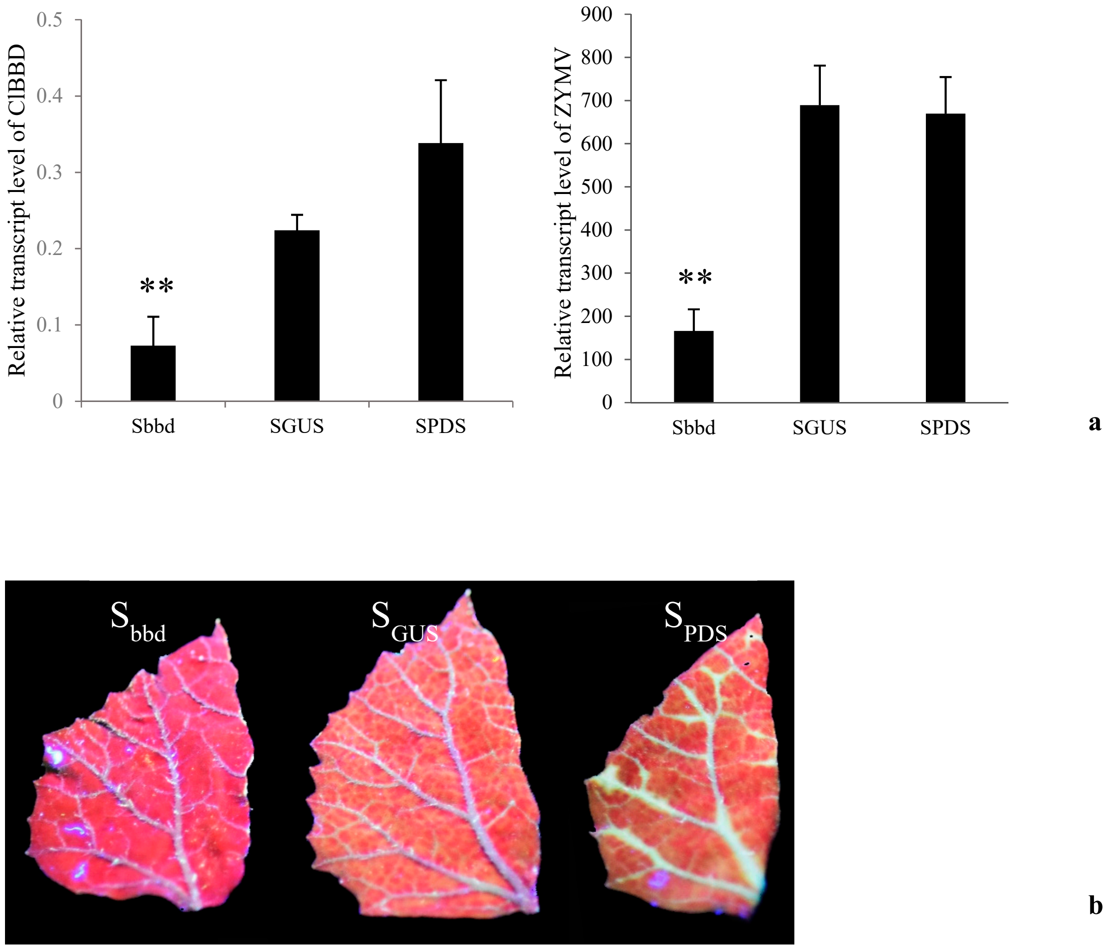

Supplementary Figure S1. The viral accumulation in melon with knockdown the expression of CmBBD by VIGS. (a) the mRNA level of CmBBD was repressed in melon at 13 days post-infiltration with S_{bbd} VIGS vectors; (b) When challenging inoculation with ZYMV-eGFP at Stage 5d, the GFP fluorescence in S_{bbd} line was weaker than those of in S_{GUS} or S_{PDS} control at 8dpi. Asterisks indicate the significant difference to S_{GUS} control; ** represent $P < 0.01$.
