# Supplementary material for: A Bifunctional Nuclease Promotes the Infection of Zucchini Yellow Mosaic Virus in Watermelon by Targeting P3
Source: Plants (Basel). 2024 Dec 6;13(23):3431. doi: 10.3390/plants13233431 (PMC11644367; doi:10.3390/plants13233431)
Supplement: Supplementary file 1 [file plants-13-03431-s001.zip › Figure S2.pdf]

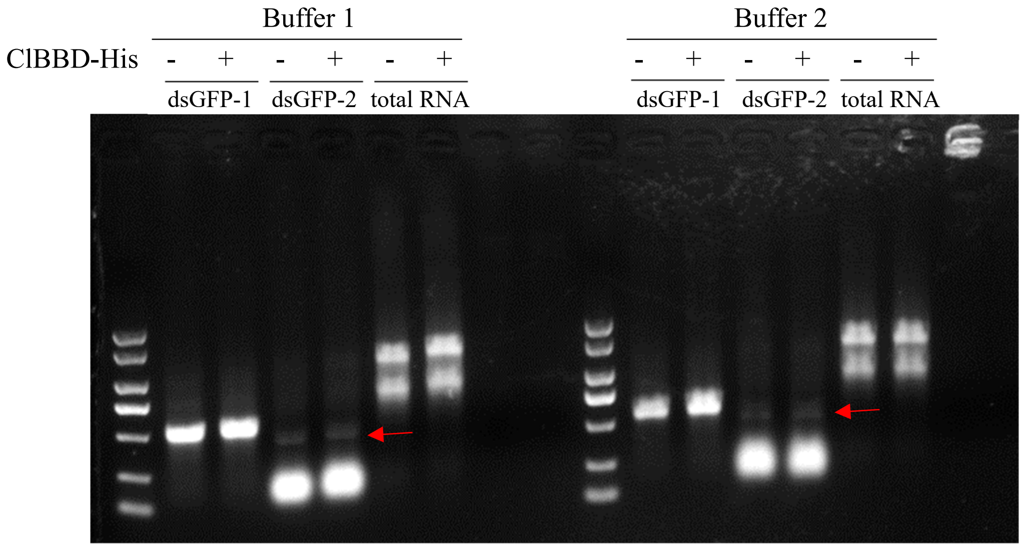

Supplementary Figure S2. Ribonuclease activity assays on dsRNA and total RNA in *vitro*. RNA were incubated in buffer 1 or buffer 2 alone (-), or with about 2.1μg purified ClBBD-His (+), at 25℃ for 2 h. DsGFP-1 was synthesized in *vitro* using MEGAscript RNAi Kit and dsGFP-2 was expressed in *E. coli*-HT115 and then was extracted using Total RNA Extraction Kit. Total RNA was extracted from the leaves of watermelon. Reaction Buffer 1 was referred to Wang et al. [28], and reaction buffer 2 was referred to Huque et al. [27]. The red arrows pointed to the dsGFP-2.
